# Thermal Conductivity of Nanoporous Materials: Where Is the Limit?

**DOI:** 10.3390/polym14132556

**Published:** 2022-06-23

**Authors:** Beatriz Merillas, João Pedro Vareda, Judith Martín-de León, Miguel Ángel Rodríguez-Pérez, Luisa Durães

**Affiliations:** 1 Cellular Materials Laboratory (CellMat), Department of Condensed Material Physics, Facultad de Ciencias, University of Valladolid, 47011 Valladolid, Spain; b.merillas@fmc.uva.es (B.M.); j.martin@cellmattechnologies.com (J.M.-d.L.); marrod@fmc.uva.es (M.Á.R.-P.); 2 University of Coimbra, Chemical Process Engineering and Forest Products Research Centre, Department of Chemical Engineering, Rua Sílvio Lima, 3030-790 Coimbra, Portugal; jvareda@eq.uc.pt; 3 BioEcoUVA Research Institute on Bioeconomy, University of Valladolid, 47011 Valladolid, Spain

**Keywords:** nanoporous materials, thermal conductivity, thermal superinsulators, aerogels, nanocellular polymers

## Abstract

Nowadays, our society is facing problems related to energy availability. Owing to the energy savings that insulators provide, the search for effective insulating materials is a focus of interest. Since the current insulators do not meet the increasingly strict requirements, developing materials with a greater insulating capacity is needed. Until now, several nanoporous materials have been considered as superinsulators achieving thermal conductivities below that of the air 26 mW/(m K), like nanocellular PMMA/TPU, silica aerogels, and polyurethane aerogels reaching 24.8, 10, and 12 mW/(m K), respectively. In the search for the minimum thermal conductivity, still undiscovered, the first step is understanding heat transfer in nanoporous materials. The main features leading to superinsulation are low density, nanopores, and solid interruptions hindering the phonon transfer. The second crucial condition is obtaining reliable thermal conductivity measurement techniques. This review summarizes these techniques, and data in the literature regarding the structure and thermal conductivity of two nanoporous materials, nanocellular polymers and aerogels. The key conclusion of this analysis specifies that only steady-state methods provide a reliable value for thermal conductivity of superinsulators. Finally, a theoretical discussion is performed providing a detailed background to further explore the lower limit of superinsulation to develop more efficient materials.

## 1. Introduction

The greatest engineering achievements of the 20th century include the automobile and airplane development, the electrification—linked to the boost of heating and cooling systems, electronic equipment (for example, the television, mobile phones, computers), and industry automation—and the internet [1]. These developments put big pressure on the energy sector, in part solved in the last century by the parallel development of petroleum and natural gas extraction and refining technologies, and nuclear technologies. While the first has limited resource availability, the second option has found many restrictions and has faced distrust from society due to the severe effects on the Planet and Humans of the hazardous wastes and catastrophic incidents [2]. Therefore, the energy availability problem crossed into this century, and it is nowadays the biggest challenge of the 21st century, along with clean water scarcity.

The energy crisis, which affects all the sectors of economy, has pushed international organizations and governments to invest a huge monetary effort in the development of renewable and clean energies, still with a slow implementation in part due to efficiency limitations. Thus, the efficient management and use of energy are always also on the table. In this regard, the insulating envelope of buildings may have a great contribution to energy saving, recognized by the EU under the Energy topic “Energy Efficiency”, which includes targets to reach “Energy-efficient buildings”, in particular nearly zero-energy buildings by 2030 [3]. In fact, buildings’ energy consumption for heating, cooling, and domestic hot water is the largest energy consumer segment in Europe, contributing to 40% of EU energy consumption and 36% of the energy-related greenhouse gas emissions. Thus, the development of more efficient insulating materials attracts the general interest of researchers, government institutions, and building-related industries, as it is a powerful tool to fight against the environmental burden and energy crisis.

Current widespread materials in building insulation include mineral/rock wool, glass wool, blown cellulose, polystyrene foams (expanded, extruded), polyurethane foams, and phenolic resin foams; in general, the last two types of foams can reach values of thermal conductivity between 20 and 30 mW/(m K), while the first options show usually values between 30 and 50 mW/(m K) [4,5,6]. This selection of insulator materials is, however, becoming narrower in some countries, due to governments restricting the use of flammable wall insulators and pushing for higher energy efficient buildings (which, amongst others, makes the walls too thick and reduces living space, due to the large thickness of the insulator required). Furthermore, these materials are also not suitable for the restoration of older or historic buildings, as façades cannot be altered, and insulators need to be placed on the outer surface of stone walls (as opposed to the inside of the wall in newer construction) [7]. It is also worth mentioning that some of these insulating solutions can also pose health threats. Glass wool can cause irritation in the eyes and respiratory track and spray foam insulation releases toxic substances when it is applied, and, if not prepared properly, these might be released for longer periods of time. Therefore, aerogels are slowly gaining a place in the market due to their very low thermal conductivity, 12–20 mW/(m K), although they still are a more expensive option [5,6].

The question now is, how much can we decrease the thermal conductivity of insulating materials? Have we reached the bottom limit? This question can be in part answered by looking at the structure of the less insulating materials and considering the thermal conductivity of air itself (26 mW/(m K), at 25 °C) [8]. In fact, Vacuum Insulating Panels (VIP) profit from the thermal conductivity of void space [4], which delivers a very good insulating efficiency, but also has issues such as loss of vacuum inside the panels due to sealing leaks. To go lower than air on thermal conductivity at ambient temperature and pressure, a very high-porosity solid material filled with air/gas is needed. It should be comprised of a 3D solid network, which can have a very low solid thermal conductivity, designed so that its porous network blocks the convective/conductive heat transfer paths of air/gas inside the pores. This is the feature of thermal superinsulating materials that can reach thermal conductivities well below that of air, this being attainable with a porous nanostructure, i.e., with average pore sizes below 66 nm (mean free path (MFP) of air molecules without constraining at ambient temperature and atmospheric pressure) [6]. The type of solids with higher thermal insulation characteristics and low costs are polymers and ceramics (especially amorphous silica), their foams being the material structures with the lowest thermal conductivity values (according to Material property charts—Ansys Granta).

The way the heat transfer crosses a porous structure is quite complex and deeply depends on the porosity value, the type of pores (cellular, random, hierarchical), and their interconnectivity (open or closed pores). The modeling of the thermal conductivity of these complex systems, in order to draw guidelines for the design of more efficient materials, is a crucial need. In addition, the reliability of the measuring methods and the applied conditions/size of the samples, either with transient or steady-state heat transfer options, is hugely important to obtain accurate values.

This review aims to provide useful insights in understanding the heat transfer phenomena in nanoporous materials and to answer the question raised in the title of this publication. The main measuring methods of thermal conductivity of insulation materials, both transient and steady-state, are described and compared, in order to raise the awareness of variability of results and preferable techniques. Then, the structures and thermal conductivity of two different nanoporous materials (nanocellular polymers and silica and polyurethane aerogels) are presented. The comparison between both materials allows a final outstanding discussion regarding thermal conductivity mechanisms, showing it as an effective strategy of correlating the structural properties of these kinds of materials and the thermal conductivity output so that this tool can be used to study the perspective of the lower limit of these superinsulators. In this way, we intend to give a contribution to the development of new and more efficient superinsulating materials.

## 2. Thermal Conductivity in Nanoporous Materials

The thermal conductivity of a nanoporous material $\left(\lambda \right)$ is given by the contribution of four terms (Equation (1)):

(1) $$\lambda =\lambda_{s}+\lambda_{g}+\lambda_{r}+\lambda_{c}$$

where $\lambda_{s}$ is the conduction through the solid phase, $\lambda_{g}$ is the conduction through the gaseous phase, $\lambda_{r}$ is the thermal radiation term, and $\lambda_{c}$ is the convection within pores. This last term can be neglected for pore sizes smaller than 2 mm [9,10]; thus, for nanoporous materials, thermal conductivity is the sum of the first three contributions.

Gaseous thermal conductivity is very reduced for cells/pores below the micron. As previously mentioned, the gas phase is confined within the small cells/pores, meaning that the gas is contained within a space of dimensions similar to their MFP (66 nm for air at atmospheric pressure and room temperature), which reduces the gas thermal conductivity, an effect known as the Knudsen effect [11]. Solid thermal conductivity is the one of the polymer skeleton. Finally, radiation contribution depends on the density and cell/pore size. The higher the relative density, the higher the fraction of absorbent material to block infrared radiation. However, when the cell/pore size decreases to the nanometric range, the wavelength of IR radiation is much larger than the cell/pore size, thus scattering is much smaller and radiation can pass through the material (Rayleigh scattering is now governing the radiation/porous structure interaction). Therefore, materials with cell/pore sizes at nanoscale have very low scattering, increasing the radiation contribution; thus, this term should be considered when the cell/pore sizes are below 50 nm and the low relative densities are low (below 100 kg/m^3^).

The following subsections describe different experimental methods to determine the thermal conductivity.

### 2.1. Measurement Techniques

Due to the high interest in synthesizing insulating porous materials, several techniques or condition adaptations focused on measuring their thermal conductivity have been developed. The main classification of these techniques is based on the regime of the heat flow, being either transient methods or steady-state methods.

#### 2.1.1. Transient Methods: Technique Description

**Figure 1:**
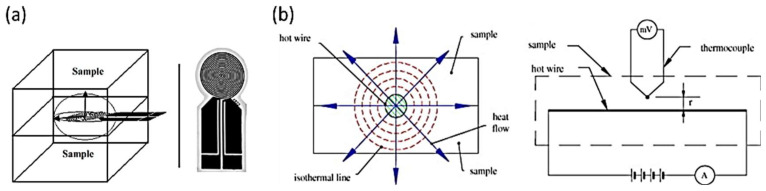
(**a**) TPS measurement scheme. Reprinted with permission from Ref. [17] Copyright © 2008 WILEY-VCH Verlag GmbH & Co. KGaA; (**b**) Hot wire method scheme. Reprinted with permission from Ref. [18] Copyright © 2007, Elsevier.

Transient measuring techniques assess the temperature response to a heat pulse, in a point in the sample with a known distance to the heat source, at defined time intervals [12,13]. Generally, they measure thermal diffusivity, are less sensitive to heat losses, generate fast results, and can be used with small samples [14]. Furthermore, these methods do not require calibration, but the sample must be in equilibrium with the environment [13]. Two main techniques are well established: the transient plane source (TPS) method and the hot wire method. A schematic representation of these measuring techniques is presented in Figure 1.

The TPS method uses a planar, circular heating source that generates a three-dimensional heat flow to the sample [15,16,17]. The heating element is placed between two identical, flat surfaced pieces of sample (Figure 1a). By measuring the temperature of the heating source over time, thermal conductivity is obtained. The standard ISO 22007-2:2015 refers to the use of this method for the determination of both the thermal conductivity and diffusivity.

In the hot wire method, an electrically heated wire, which operates as a linear heat source, is inserted into the sample and the temperature increase in the latter is measured at a specific distance from the wire (alternatively, the temperature of the wire may be measured over time) [15,16]. The heat flows radially from the wire to the sample [18]. This method has the standard ASTM C 1113 associated with it.

The mathematical theory for these techniques has long been detailed [19,20].

#### 2.1.2. Steady-State Methods: Technique Description

The fundamentals of the steady-state methods are based on establishing a temperature difference between both sides of the sample that does not change over time. In this way, a temperature gradient is promoted throughout the sample until reaching the steady-state condition in which the heat flux is constant and the temperature along the specimen is the same. Therefore, the Fourier’s law for the steady-state method poses a one-dimensional problem:

(2) $$\lambda =\frac{Q\cdot d}{A\cdot \Delta T}$$

where *Q* is the heat flow throughout the specimen (W), *A* is the area in which the heat flow is being transmitted (m^2^), *d* is the sample thickness (m), and Δ*T* the temperature difference between the sample surfaces (K).

One of the main drawbacks of this method is the long time required to reach the steady-state condition, longer than for transient methods, and the large sample size required to carry out the experimental measurements. The latter is limitative for research samples at lab scale but is related to the assumption of unidirectional heat flow in the sample, which imposes nearly null losses through the sample boundaries.

**Figure 2:**
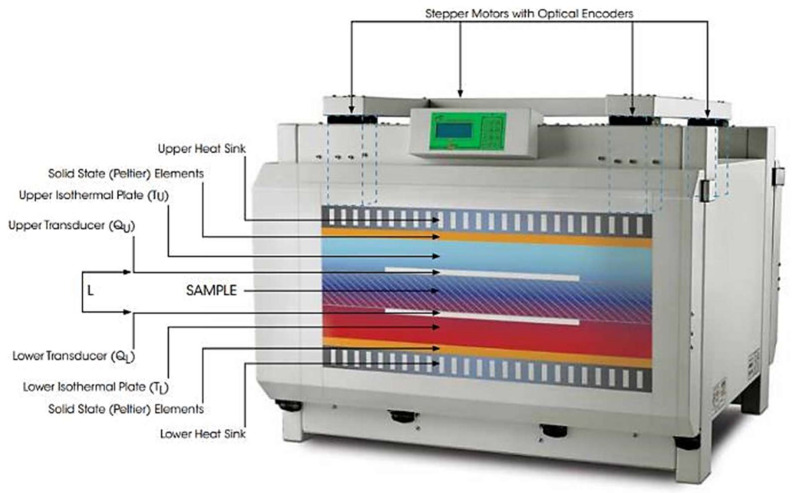
Heat flow meter measurement scheme [26].

Nevertheless, the advantages are rather relevant, since the temperature distribution is not time-dependent (which occurred for transient methods) and the equation to solve is straightforward providing a high accuracy [21]. Several techniques follow the steady-state method with the corresponding measuring international standards, such as the heat flow meter (single-specimen type) (ISO 8301:1991) (ASTM C518) [22] (UNE-EN 12667) [23], “cur bar” methods (using two reference bars) (ASTM D5470) or guarded hot plate (double-specimen type) (ISO 8302) [24] (ASTM C177) [25]. These standards establish a large sample size as a requirement for measuring. A schematic representation of the heat flow meter technique is presented in Figure 2.

#### 2.1.3. Empirical Comparison between Transient and Steady-State Methods

The effectiveness of the different thermal conductivity measurements depends on several characteristics of the material structure such as density, the size, and distribution of the solid constituents and the pores or voids present on the specimen [27]. Additionally, there are several factors to consider when selecting a technique for measuring thermal conductivities as the type of material, the measurement time, temperature range, measurement accuracy, as well as the range of thermal conductivities. These factors, together with some key aspects, will be analysed in this section for the transient and steady-state methods (Table 1).

On the one hand, the main difference lies in the fundamentals, since the transient methods are pulsed power techniques in which an electric periodical heating current is used, whereas the power input does not change with time in the steady-state methods. The latter are absolute techniques in which the heat flow rate is directly measured, while non-steady techniques calculate this heat flow through the measurement of the temperature gradient and the electrical resistance of the heater, therefore, being derivative techniques [28]. The fact that transient methods are typically indirect adds further uncertainties affecting the measurement. These uncertainties have a relevant effect for very low thermal conductivities in which a variation of few milliwatts per metre kelvin is crucial for the description of insulating materials [29]. Regarding the time consumption of each method, it is significantly longer for the steady-state ones since reaching the equilibrium temperature is required, while non-steady techniques are relatively fast [30].

On the other hand, aspects related to the sample characteristics also play a significant role. For instance, one of the main drawbacks of the steady-state techniques resides in the relatively large samples needed for carrying out the measurements (typically above 5 × 5 cm^2^). For this reason, several works have focused their efforts on developing new procedures for measuring insulating samples with a smaller size. Jannot et al. [31,32] reported a centered hot plate method for measuring with a high precision the thermal conductivity of small insulating samples (3–9 mm of thickness and a diameter of 15 mm) with thermal conductivities in the range 0.014–0.2 W/(m K). Miller et al. [33] developed a hot-plate method for measuring insulating samples (2.5 mm thickness and 20 mm of diameter).

Due to the differences between both type of methods, a comparison based on experimental measurements has been carried out. The used nanoporous samples are silica aerogel composites which show thermal conductivities comprised in a wide range, and a nanocellular poly(methyl methacrylate) (PMMA). The experimental values are gathered in Table 2 as well as the bulk density of the specimens. The values measured by the transient method (TPS) were obtained by a HDMD (Hotdisk) and those for the steady-state by a heat flow meter (Fox 314, TA Instruments) in which an external heat flux sensor (gSKIN® XM 27 9C, greenTEG AG) has been coupled [34]. The selected samples present densities between 78 and 403 kg/m^3^ and thermal conductivities between 28.1 and 92.8 mW/(m K) (TPS method) and between 11.5 and 71.3 mW/(m K) according to the steady-state method. There exists a non-negligible increase of the values when the samples are measured by the TPS method.

**Figure 3:**
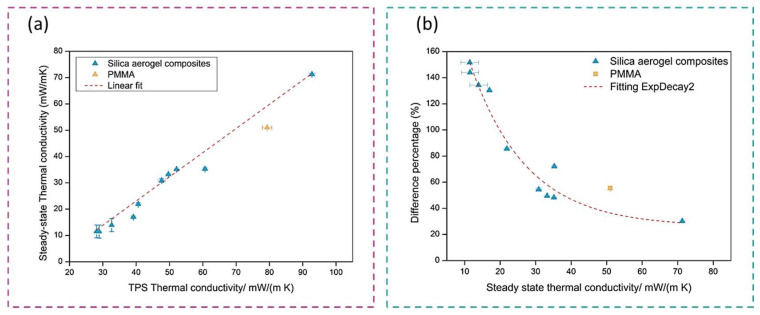
(**a**) Correlation between steady-state and TPS thermal conductivity experimental values; (**b**) difference percentage between TPS and steady-state methods as a function of the thermal conductivity.

The TPS and steady-state measuring methods were compared (Figure 3a) and the obtained tendencies predicted by both techniques are the same, showing a good correlation between them.

In order to assess the difference between these methods and its dependence with the thermal conductivity value, the percentage of the difference between both of them has been calculated:

(3) $$\%\;\mathrm{difference}=\frac{\left(\mathrm{TPS}\;\mathrm{value}-\mathrm{Steady}\;\mathrm{state}\;\mathrm{value}\right)}{\mathrm{Steady}\;\mathrm{state}\;\mathrm{value}}\times 100$$

The difference percentage in relation to the thermal conductivity of the steady-state method is plotted in Figure 3b. As expected, the increase in the thermal conductivity obtained with the TPS method has a stronger relevance for materials with the lowest thermal conductivity (below 20 mW/(m K)), reaching differences above 140%. For thermal conductivities between 20 and 40 mW/(m K), the difference between both methods decreases but it is still high (from 40 to 80%). Nevertheless, when measuring thermal conductivities in the order of 70 mW/(m K), the difference between both methods is minimized, reaching differences *ca.* 30%. It must be noticed that the latter value continues to be significantly high even though the sample shows a poor insulating performance.

For these reasons, transient methods are valid to compare samples, but the steady-state methods provide the most accurate values when measuring the thermal conductivity of nanoporous materials.

### 2.2. Thermal Conductivity of Nanocellular Polymers

As it was previously stated, different nanoporous structures will be analyzed with the aim of comparison. Nanocellular polymers with nanoporous structure have been shown to present a thermal conductivity that is not as reduced as that of aerogels (materials presented in the Section 2.3). In the following paragraphs, an in-depth description of the structural features of these materials and their thermal conductivity is given.

#### 2.2.1. Nanocellular Polymers

Nanocellular polymers are defined as cellular polymers with the cell size below the micron, and cell density (number of cells per unit volume) higher than 10^13^ cells/cm^3^ [35,36]. These materials have grabbed the attention of the scientific community due to the combination of properties they could achieve. Nanocellular polymers have been proved to present enhanced mechanical properties in comparison with cellular polymers with larger cell sizes; for cell sizes below 50 nm, they somehow preserve the transparency of the precursor solid, and they present low gaseous thermal conductivity due to the presence of the Knudsen effect, which could lead to a reduced total thermal conductivity.

Nanocellular polymers can be produced through different techniques; however, the most promising one, to date, for the production of thick samples is gas dissolution foaming [37]. Gas dissolution foaming is a physical foaming process consisting of four steps: saturation, depressurization, foaming, and stabilization. During saturation, the gas, usually CO_2_, is dissolved into the polymer at certain conditions of saturation pressure $\left(P_{\mathrm{sat}}\right)$ and temperature $\left(T_{\mathrm{sat}}\right)$. The gas dissolving inside the polymer decreases the initial glass transition temperature $\left(T_{g}\right)$ up to the effective glass transition temperature $\left(T_{g_{\mathrm{eff}}}\right)$. When the polymer does not admit more gas, saturation time $\left(t_{\mathrm{sat}}\right)$, at the fixed saturation conditions, the polymer is said to be saturated. The pressure is then released, during the depressurization step, at a high depressurization velocity $\left(v_{\mathrm{des}}\right)$. This pressure decay leads to a thermodynamic instability leading to the production of small gas clusters known as nucleation points. Simultaneously to nucleation, the gas diffuses out of the polymer due to the pressure difference between dissolved gas and the atmosphere. After the desorption time $\left(t_{\mathrm{des}}\right)$, the foaming step is carried out, the polymer is heated at a foaming temperature $\left(T_{f}\right)$ above its $T_{g_{\mathrm{eff}}}$ during a particular foaming time $\left(t_{f}\right),$ and nucleation points grow into cells. Finally, the material is stabilized at a temperature below the $T_{g_{\mathrm{eff}}}$ preventing the degeneration of the cellular structure. If the depressurization takes place at a temperature above the $T_{g_{\mathrm{eff}}}$, nucleation and growth coexist, a process known as one-step foaming [38].

As previously described, nanocellular polymers require the presence of a high number of tiny cells. Two different approximations can be followed to produce them, homogeneous nucleation and heterogeneous nucleation. In heterogeneous nucleation, a second phase is intentionally added to act as preferential sites for nucleation, the number and size of cells being determined by this second phase. In homogeneous nucleation, this second phase is not present, and nucleation is mainly controlled by the production parameters [39,40].

**Figure 4:**
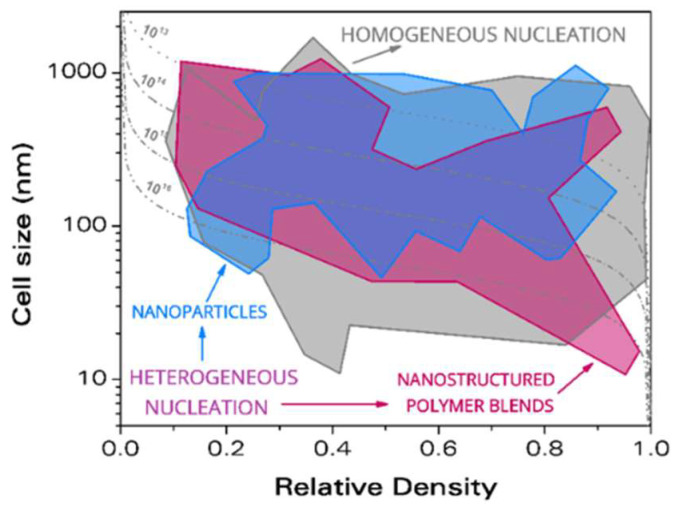
Comparison between the materials that have been produced taking advantage of homogeneous and heterogeneous nucleation (from reference [50]).

Both approximations have led to the production of nanocellular materials with a wide variety of cell sizes and relative densities as can be seen in Figure 4. Achieving low thermal conductivities relies on producing materials with small cell sizes combined with small relative densities (see Section 3). The region of the lowest achieved cell size combined with the lowest relative density is covered by materials produced through homogeneous nucleation. Martin-de León et al. reported the nanocellular polymer with the smallest cell sizes in the literature produced from PMMA, with cell sizes below 20 nm and relative density below 0.4 [41,42]. Guo et al. also reported some interesting data. They produced nanocellular polycarbonate with cell sizes of 21 nm and relative densities of 0.56 [43]. By using polysulfone (PSU) and polyphenylsulfone (PPSU), they presented materials with cell sizes of 22 and 26 nm with respective relative densities of 0.84 and 0.59 [44,45]. It should be mentioned that the materials with the lowest relative density and cell sizes below 100 nm are presented by Costeux et al. with PMMA copolymer (PMMA-co-EA), reporting a relative density of 0.18 with 80 nm of cell size [46], while Martin-de Leon et al. reported 75 nm with a relative density of 0.24 [41]. Regarding materials produced through heterogeneous nucleation, it is worth mentioning those produced by Wang et al. using PMMA + Thermoplastic polyurethane (TPU), with relative densities as low as 0.13 and cell sizes up to 170 nm [47]. With the same system, Bernardo et al. reported foamed beads with a relative density of 0.14 and cell sizes below the micron [48]. Costeux et al. produced PMMA with polyhedral oligomeric silsesquioxanes (POSS) as nucleating agent, with a cell size of 65 nm and relative density of 0.26 [24]. Other interesting materials are those produced by Bernardo et al. through the addition of the copolymer MAM (poly(methyl methacrylate)-poly(butyl acrylate)-poly(methyl methacrylate) to PMMA leading to cell sizes of 582 nm and a relative density of 0.162 [49].

However, the bottom left region in the map of Figure 4 has not yet been achieved with these nanocellular polymers, which hinders us from achieving the desired extremely low thermal conductivity. This region was filled by aerogels, and it is possible to gain insight on how to decrease the thermal conductivity of insulators by studying aerogel systems.

#### 2.2.2. Thermal Conductivity in Nanocellular Polymers

Although low thermal conductivity in nanocellular polymers is claimed to be one of its most interesting properties, data regarding experimental values are really scarce.

Table 3 shows the most significant experimental values that can be found in the literature for bulk nanocellular polymers. All the reported values are for nanocellular materials based on PMMA produced both through homogeneous (PMMA) and heterogeneous nucleation (PMMA/MAM, PMMA/TPU and PMMA/SEP). In addition, all the thermal conductivity values are determined through the TPS technique.

Notario et al. reported a range of cellular materials produced with heterogeneous nanocellular PMMA/MAM with cell sizes from the micro to the nanoscale. The material with the lowest density (420 kg/m^3^) presents a cell size of 950 nm and a total thermal conductivity of 83.7 mW/(m K), which is at the same time the lowest value reported in said work. The material with the lowest cell size of 94 nm and density of 710 kg/m^3^ has a thermal conductivity of 104.3 mW/(m K) [11].

The lowest value of Table 3 is reported by Wang et al. with a thermal conductivity of 24.8 mW/(m K) for a nanocellular PMMS/TPU material with 205 nm of cell size and a bulk density of 153 kg/m^3^. This result corresponds also to the lowest bulk density materials of Table 3. However, the same authors have recently theoretically proved that such low thermal conductivity cannot be reached with the reported characteristics of the material [51]. Martin-de León et al. produced nanocellular materials by means of homogeneous PMMA with cell sizes of 225 nm and 25 nm and a range of densities. For materials with cell sizes of 225 nm, the lowest thermal conductivity of 58.8 mW/(m K) corresponds to the minimum relative density of 249 kg/m^3^. On the other hand, materials with 25 nm of cell size present a minimum value of 72.4 mW/(m K) for 415 kg/m^3^ of density.

Bernardo et al. have studied bimodal cellular materials produced through the addition of MAM and sepiolites to PMMA. The produced materials present an important volumetric fraction of cells in the nanometric range. The lowest conductivity for PMMA with MAM is 70 mW/(m K) corresponding to a cellular structure with 258 nm of cell size with a 15% of cells with 2.9 microns. With the addition of sepiolites, a value of 80 mW/(m K) is obtained for a bimodal cell size of 296 nm and a 43% of cells of 2.1 µm.

**Table 3:** Bulk density, cell size, and thermal conductivity of bulk nanocellular polymers in the literature.

Material	Bulk Density/ kg/m^3^	Cell Size/ nm	Thermal Conductivity/ mW/(m K)	Ref.
PMMA/MAM	420	950	83.7	Notario B. et al., 2015 [11]
	570	820	107.2	
	490	300	88.4	
	470	290	88.4	
	510	235	92.5	
	480	220	90.0	
	690	200	101.5	
	600	150	94.8	
	650	130	94.7	
	710	94	104.3	
PMMA/TPU	165	930	36.9	Wang G. et al., 2017 [47]
	153	205	24.8	
PMMA	486	225	87.5	Martín-de León J. et al., 2019 [52]
	403		79.3	
	320		71.3	
	249		58.8	
	605	25	97.0	
	546		89.6	
	522		87.2	
	510		83.9	
	474		79.3	
	451		76.9	
	415		72.4	
PMMA/MAM ^(a)^	415	Bimodal: 258 nm + 2.2 µm (15%)	83.0	Bernardo V. et al., 2019 [53]
	320	Bimodal: 276 nm + 2.1 µm (14%)	70.0	
PMMA/SEP ^(a)^	451	Bimodal: 260 nm + 2.9 µm (30%)	92.0	Bernardo V. et al., 2019 [53]
	0.29	Bimodal: 296 nm + 2.1 µm (43%)	80.0	

^(a)^ Bimodal cellular materials; the volumetric fraction of microcells is in brackets.

**Figure 5:**
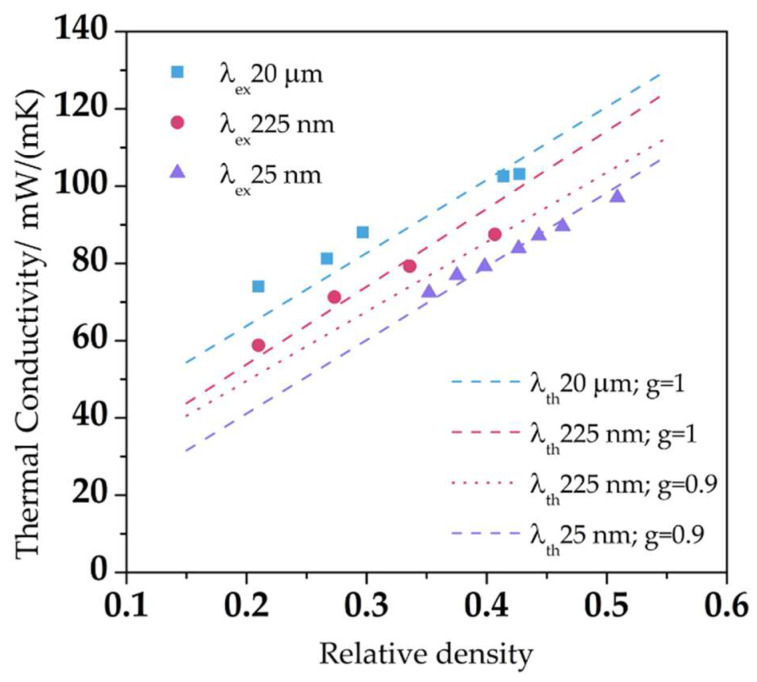
Experimental (points) and theoretical (dashed lines) thermal conductivities for nanocellular PMMA with different cell sizes as a function of the relative density (Adapted from reference [52]).

The reported values have proven to be interesting issues from an academic point of view, such as the experimental validation of the Knudsen effect in nanocellular polymers, or the experimental demonstration that materials with the same relative density but cell sizes of 225 nm present lower thermal conductivity than those microcellular ones. In addition, if the cell size is again reduced to 25 nm, the value is further reduced. As can be seen in Figure 5, the thermal conductivity is decreasing as the relative density reduces, but values for smaller cell sizes are always smaller [52].

However, and still taking into account the reduction in thermal conductivity given by measuring through the steady-state method (see Section 2.1.3), the presented values are far from those presented by aerogel insulators. In fact, the lowest thermal conductivity reported in the literature for cellular PMMA is 32 mW/(m K) and corresponds to cells around 150 µm and low relative densities [51].

Nanocellular polymers are therefore far from being included within the definition of thermal superinsulators (materials that feature conductivities below that of the air), in which the aerogels of the following section are for sure included.

### 2.3. Thermal Conductivity of Aerogels

In this section, a summary of the more relevant results of silica and polyurethane aerogels regarding their insulating performance is carried out.

#### 2.3.1. Silica Aerogels: Effect of Structural Properties on the Thermal Conductivity

Silica aerogels were the first aerogels produced [54,55] and still account for ca.20% of the aerogel research today [56]. These extremely lightweight materials have superinsulation characteristics (typical thermal conductivity of ~15 mW/(m K) in a pure silica aerogel [57]) that drive their market applications, although other applications are becoming more frequent [58,59,60,61,62]. The aerogel market is projected to grow [63], fostered by the demand for energy efficient buildings and the need to halt CO_2_ emissions due to heating. In buildings, aerogels can be applied in many forms and in different construction materials: as wall insulators, in fenestration, and incorporated in cements and plasters [5]. Furthermore, they can be applied in older buildings, improving their energy efficiency without compromising the aesthetics of façades [64,65]. However, their high cost and difficult handling (silica aerogels are known for being fragile and shedding particles) are still inhibiting their ubiquitousness.

Researchers continue the improvement of silica aerogels in order to further decrease their thermal conductivity, improve handling, machinability, and safe use. In this section, silica aerogels with super insulating capabilities (*k* < 25 mW/(m K)) are discussed and presented in Table 4. Silica-based aerogels with higher thermal conductivity also appear often in the literature, up to ca. 30 mW/(m K), normally as a result of the higher fraction of macropores originated by the organic moieties in the silica backbone (when using the co-precursor method) or/and by the presence of fibers as reinforcement.

Numerous works compiled in Table 4 report the development of silica–fiber aerogel composites [65,67,68,70,74,75,79]. In fact, this strategy is very efficient in making larger aerogels that have better machinability. Some works, however, report silica aerogels without reinforcement (polymers or fibers) [69,71,72,76,78]. Regarding the processing, the majority of works compiled in Table 4 were prepared with TEOS as silica source (or as a co-precursor) [66,67,69,71,73,74,75,76,77,78,79,80,81]. Ambient pressure drying (i.e., evaporation of the solvent at ambient pressure) is also becoming more common in superinsulating materials [66,69,72,73,74,77]. This comes to show that silylation strategies (e.g., with hexamethyldisilizane or hexamethyldisiloxane) are efficient in producing materials with aerogel-like properties, as it was discussed earlier [82].

The microstructure of the aerogel determines its total thermal conductivity, as heat can be conducted in the solid matrix, by the air in the pores if these are larger than the air’s MFP, and due to radiation [83]. Moreover, thermal coupling effects are also shown to be present [84] in aerogels and can be affected by the backbone connectivity [85]. Thus, decreasing the pore size down to the mesopores range is essential to minimize the thermal conductivity. However, it is pertinent to mention how the pore volume/size is reported because the commonly employed BJH theory to assess the pore size distribution does not apply well to aerogels due to the predominance of non-cylindrical pores. Thus, the average pore size presented in Table 4 is obtained in different ways, which may justify some discrepancies in the thermal conductivity of materials with similar bulk density. On the other hand, on the work by Stojanovic [73], the TEOS derived silica aerogel powder has larger pores than the waterglass derived one (17.6 vs. 12.9 nm) and different bulk densities (70 vs. 110 kg/m^3^, respectively), but the obtained conductivity is very similar in both materials.

By analyzing Table 4, only a handful of works report thermal conductivities as low as the typical conductivity (15 mW/(m K)) for pure silica aerogels. As we discuss in this work (Section 2.1.3), the measuring technique for thermal conductivity also affects the value obtained. Even in the same method, there may be differences, for example in the work by Ghica et al. [66], measuring with different size sensors (hence, different size samples) in a HotDisk equipment (TPS method) resulted in different thermal conductivity values for same sample replicas. In this method, the thermal conductivity of insulating samples tends to decrease with the increase of the sensor size. In most of the works compiled in this section, the thermal conductivity was estimated by the guarded hot plate method (steady-state method). As pure silica aerogels are very fragile, brittle, and are not moisture resistant, the majority of the works compiled are organically modified silica aerogels or aerogel composites. As it can be seen, these types of materials are now reaching extreme thermal insulating performance. However, a thermal conductivity inferior to 25 mW/(m K) is still uncommon in the literature.

#### 2.3.2. PU-Aerogels: Effect of Structural Properties on the Thermal Conductivity

Organic aerogels are considered as potential alternatives to silica aerogels owing to their high impact strength [86]. Therefore, researchers have focused their attention on improving their insulating performance in order to reach similar values to those of silica aerogels. The most interesting matrixes are based on polyisocyanurate or polyurethane, two matrixes that have been commonly used for insulation in the construction sector in the form of foams [87,88,89,90]. Nevertheless, the thermal conductivity reached by these polymeric foams is far from the targeted insulating performance. Thus, in recent years, aerogels based on polyurethane and polyisocyanurate have been developed allowing for obtaining thermal conductivities below air (26 mW/(m K)) and, therefore, being considered superinsulating materials. The structure of these materials is similar to that of the silica aerogels, being formed by small spherical particles in the nanometric scale and branched mesopores. The small size of these features has recently led to the obtention of the first polyisocyanurate-polyurethane (PUR-PIR) aerogels presenting transparency [91,92]. The main research showing low values of thermal conductivity for these aerogels is gathered in Table 5.

Interesting insulating abilities have been achieved, matching the performance of silica aerogels. The lowest value of 15 mW/(m K) was reached in 1998 by Biesmans et al. [20]. Since then, several attempts to reduce this value were made reaching low thermal conductivities of 17 and 19 mW/(m K) by Diascorn et al. and Zhu et al., respectively. Recently, a value of 12 mW/(m K) has been obtained by Merillas et al. [91]. It is noticeable that the pores forming these aerogels are mainly mesopores, thus contributing to reducing the gaseous contribution to the effective thermal conductivity. In some cases, these pores are larger than 100 nm ([92,95,96,98]) owing to the difficulty to measure the pore size in a system with interconnected pores and being of few nanometres.

Although the scarce literature in this field, polyurethane and polyisocyanurate aerogels are promising materials for thermal insulation applications. Despite thermal conductivity of solid silica and solid polyurethane being very different (1310 mW/(m K) [99] and 260 mW/m K respectively [100]), the final thermal conductivity of both aerogels (Table 4 and Table 5) reaches a nearly similar lowest value. This means that the key point to obtaining such small values is in the production of this aerogel-type materials, which leads to particular structural features, as will be discussed in the next section.

## 3. Modeling Thermal Conductivity of Nanoporous Materials 

In the following sections, the different heat transfer mechanisms will be analyzed, leading to the key factors explaining the differences between both studied structures, nanocellular polymers and aerogels.

### 3.1. Radiation Term

**Figure 6:**
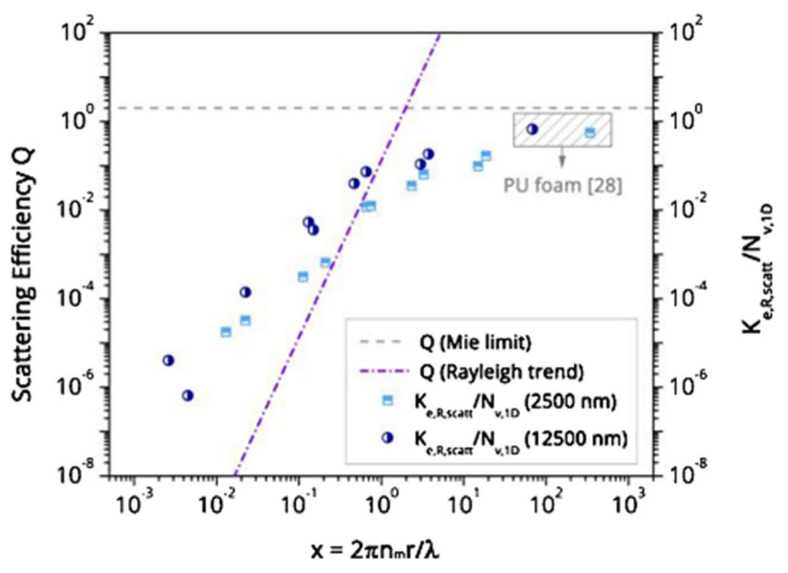
Right axis: Scattering efficiency *Q* for a Rayleigh-like behaviour and Mie limit as a function of the size parameter *x*. Left axis: Scattering extinction coefficient normalized by the 1D cell density as a function of the size parameter *x.* (Reprinted with permission from Ref. [101], Copyright © 2020, Elsevier).

The radiation term accounts for the infrared radiation transmitted through the nanoporous structure. IR radiation can be both absorbed by the solid phase or scattered by both phases. The absorbed radiation depends on the type of solid phase and the amount of the same. The scattering is mainly determined by the pore size. When the pore size becomes smaller than a tenth of the wavelength, the scattering becomes smaller and therefore transmittance increases, raising the radiation term (see Figure 6) [101].

This radiation term can be calculated through the Rosseland Equation (4):

(4) $$\lambda_{r}=\frac{16n^{2}\sigma T^{3}}{3K_{e,R}}$$

where n is the refractive index, σ is the Stefan–Boltzman constant, T is the temperature, and $K_{e,R}$ is the Rosseland extinction coefficient.

Radiation conductivity has been proved to have an important effect both in aerogels, with high transmittances in frequencies where the solid materials do not have an absorption band [102], and in nanocellular polymers, where this term has been recently measured [101]. Figure 6 represents the scattering efficiency for a sole pore as a function of the factor $x=2\pi n_{m}r/\lambda$, with $n_{m}$ the refractive index of the surrounding medium and r the radius of the scattering center. It can be seen how it decreases for small radii.

Both compared materials (aerogels and nanocellular polymers) will therefore behave similarly regarding the radiation term.

### 3.2. Gaseous Thermal Conductivity

Conduction through gaseous phase depends on the effective gaseous thermal conductivity $\lambda_{g}^{\prime }$ and on the relative amount of gaseous phase as follows (Equation (5)):

(5) $$\lambda_{g}=\lambda_{g}^{\prime }\left(1-\rho_{r}\right)$$

**Figure 7:**
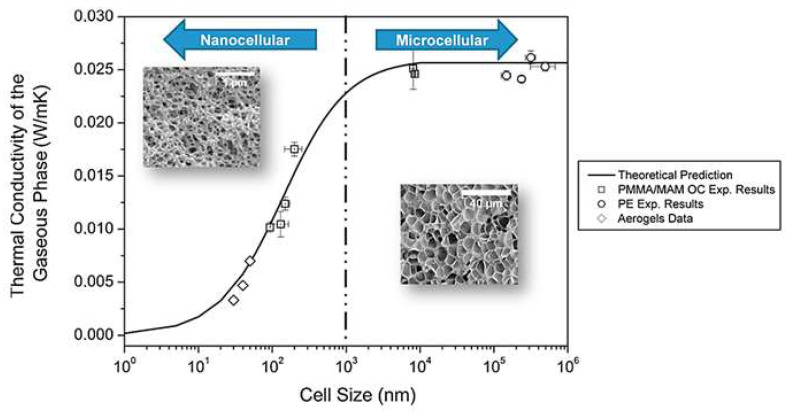
Gaseous thermal conductivity as a function of the cell size for different systems (Reprinted with permission from Ref. [11], Copyright © 2015, Elsevier).

When working with conventional cellular materials, the gaseous thermal conductivity is considered as the thermal conductivity of the gas inside the cellular material under the working conditions (usually air with thermal conductivity of 26 mW/(m K) at room temperature and atmospheric pressure). However, nanoporous systems present pores comparable to the MFP of air molecules (70 nm for air normal conditions [103]) leading to what is called confinement of the gaseous phase within pores. This brings a reduction of the gaseous thermal conductivity along the pores. When the pore size is similar to the MFP of the air molecules, they are more probably colliding with cell walls delimiting the pore than among them. This is known as Knudsen effect (Figure 7) and results in a strong reduction of the effective gaseous thermal conductivity described through Equation (6):

(6) $$\lambda_{g}^{\prime }=\frac{\lambda_{g0}^{\prime }}{1+2\beta \frac{l_{g}}{\phi }}$$

where $\lambda_{g0}^{\prime }$ is the thermal conductivity of the gas inside the pores, β is a factor given the transfer of energy between gas molecules and the porous structure (1.64 for air [104]), $l_{g}$ the mean free path, and ϕ the average pore size. The ratio between the mean free path and the cell/pore size is known as the Knudsen number $\left(K_{n}\right)$.

According to the literature, the reduction of the gas thermal conductivity starts to take place when the Knudsen number is higher than 0.01, which means for cell/pore sizes smaller than 100 times the mean free path, $\phi <100\;l_{g}$ [105]. Taking this into account, the Knudsen effect should be considered for pores smaller than seven microns independently of the used material. As it can be seen in Figure 7, this effect is indeed valid for both nanocellular polymers in Section 2.2 and aerogels in Section 2.3. Figure 7 represents Equation (6) as a function of the cell size. When the pore size is higher than 7 µm, the gaseous conductivity is that of the air, decreasing this value significantly as the porous size reduces. Thus, when the pore size is smaller than 100 nm, the gaseous thermal conductivity is below 8 mW/(m K), a value that decreases up to 5 mW/(m K) for porous diameters as small as 5 nm.

Both nanoporous structures, aerogels and polymeric materials, have been proved to present cell sizes below 7 micrometres and as small as 14 nm in the case of nanocellular polymers [42] and 7 nm regarding silica aerogels, thus effective thermal gaseous conductivity, may reduce similarly for both structures. However, as previously mentioned, relative density for nanocellular materials with cell sizes smaller than 100 nm has not been reduced below 0.18, while the minimum value reported for silica aerogels may be below 0.1. This leads to a small difference of some tenths of mW/(m K), in this second term between both structures.

### 3.3. Solid Thermal Conductivity

Conduction through the solid phase depends on the proportion of the solid, which means the relative density, the thermal conductivity of the matrix ( $\lambda_{s}^{\prime }$), and the structural factor g (Equation (7)):

(7) $$\lambda_{s}=g\lambda_{s}^{\prime }\rho_{r}$$

The g factor accounts for the heat conduction through the solid porous structure and how the characteristics of the porous structure, such as the geometry of the pores, open or cell structure, or continuity of the solid structure affects the final solid thermal conductivity.

Thermal conductivity through the solid phase can be understood as the propagation of lattice vibrations. For crystalline materials, phonons quantify those vibrations. The same concept can be used for amorphous materials (the definition of phonons is unclear for amorphous polymers; however, this concept is commonly used in polymer science). Covalent and electrostatic interactions in polymers affect the overall thermal transport. Short crosslinkers were shown to enhance interchain thermal conduction through non-covalent interactions; thus, cross-linking can become an useful strategy to control the thermal conductivities of polymeric materials through changes in the cross-linker chains length [106]. As particles obey the Bose–Einstein statistics, the thermal conductivity through the solid will be given by Equation (8) [100]:

(8) $$\lambda =\frac{1}{3}\underset{i}{\sum }\int C_{i}\left(\omega \right)v_{i}l_{i}\left(\omega \right)d\omega \;$$

$C_{i}\left(\omega \right)d\omega$ is the heat capacity contribution of phonons with a polarization i and frequency ω, $v_{i}$ the phonon velocity and $l_{i}\left(\omega \right)$ the phonon MFP. The MFP is the distance a phonon can travel before colliding, which means before suffering scattering. Increasing the scattering events along the solid material will therefore decrease the thermal conductivity through the solid phase.

In addition, the size of the solid region in which phonon transport is taking place is also important. If the material size is comparable to the MFP, phonon transport is limited reducing the thermal conductivity. This is called size effect, and the thermal conductivity as a function of the sample thickness (L) is given by Equation (9) [107]:

(9) $$\lambda \left(L\right)=\lambda_{\mathrm{bulk}}{\left(1+\frac{4\;\mathrm{MFP}}{3L}\right)}^{-1}$$

$\lambda_{\mathrm{bulk}}$ is the conductivity of the solid polymer with infinite dimensions. The phonon mean free path of the bulk solid can be calculated as Equation (10):

(10) $$\mathrm{MFP}=\frac{3\lambda_{\mathrm{bulk}}}{C_{v}v_{g}}$$

$C_{v}$ is the specific heat per unit volume, and $v_{g}$ is the mean group velocity of phonons.

All of this can explain one of the main differences between aerogels and nanocellular polymers. *g* factor has been reported to present values from 0.3 to 1 in cellular polymers, while in aerogels, values as low as 0.005 can be found, leading to a really small solid thermal conductivity.

On the one hand, aerogels are known for presenting a backbone structure with multiple discontinuities and small contact points. This structure enhances the phonon scattering. On the other hand, the solid regions in aerogels can be limited to a few nanometres. According to Equation (10), this leads to size effect and a reduction in the solid thermal conductivity [108]. On the contrary, nanocellular polymers present an interconnected solid phase, with no hindering for thermal conduction. Additionally, the MFP in polymers ranges from 1.5 to 10 nm [109], and the smallest cell walls reported in the literature are 20 nm in thickness, far away from these values. No scattering or size effect in nanocellular polymers leads to a much higher *g* factor than those published for aerogels, meaning that a nanocellular polymer with a similar porous size and relative density of an aerogel presents a thermal conductivity through the solid phase at least six times higher.

As it was previously said, there exist some differences between the analysed aerogels. The solid thermal conductivity for both of them will depend on the thermal conductivity of the solid matrix and, as discussed, on the size of the particles or their bonding nature, which can lead to different phonon scattering. The key point is that this phonon scattering can be therefore tailored for aerogels, while it is not present in nanocellular polymers.

### 3.4. Future Perspectives

**Figure 8:**
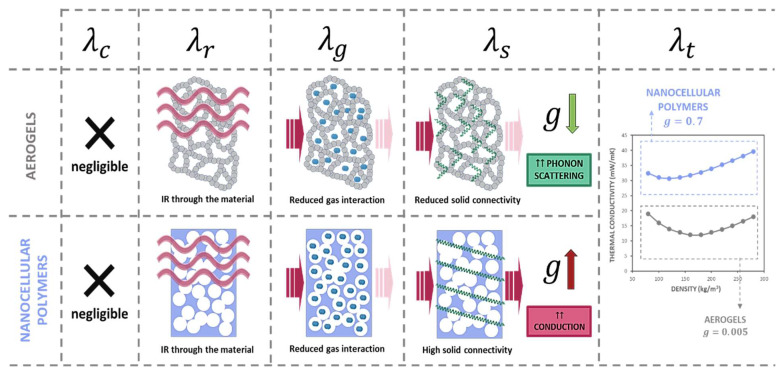
Heat transfer mechanisms in aerogels and nanocellular polymers. While both systems behave similarly in terms of radiation and conduction through the gas phase, there is a huge difference in the conduction through the solid material due to the intrinsic configuration of the solid phase. Nanocellular polymers show a connected solid phase, and as a result, higher conductivity.

Differences between both nanoporous structures are summarized in Figure 8. As it can be seen, both structures behave similarly in terms of conduction and radiation. Although both materials present the Knudsen effect, the gaseous phase proportion is in general higher for aerogels, leading to a smaller gaseous thermal conductivity. Finally, the higher difference can be attributed to the solid thermal conductivity. The discontinuity of the aerogel solid-phase leads to phonon scattering and size effect reducing this term in comparison with the one of nanocellular polymers.

Although it has been proved that up to now the thermal insulation performance is better for aerogels, they present some drawbacks that are overcome with nanocellular polymers. The aforementioned aerogels have, in general, poor mechanical properties and their price is high due to the complexity of their production processes. In addition, these processes are not environmentally friendly, in some cases, when using certain precursors and due to the possible presence of hazardous solvents [110].

On the contrary, nanocellular polymers in the literature are mainly PMMA based, which is a recyclable material. In addition, the production method is a sustainable process and the use of CO_2_ allows not leaving any hazardous solvents during the process [39]. They have also been proved to present very promising mechanical properties [111,112]. Improving thermal performance in nanocellular polymers needs both a reduction in relative density and the hindering of phonon diffusion. Regarding the bulk density, it is theoretically proved that closed-cell materials cannot present cell sizes below 10 nm together with low densities. However, open-cell material could lead to combining cell sizes of 20 nm with porosities over 0.9 [113]. Regarding the reduction in solid thermal conductivity, strategies to enhance phonon scattering should be studied.

Considering the discussion in this review, both types of materials could take advantage of the knowledge in the other one. In fact, the possibility of combining both materials to generate the superinsulators of the future opens up. 

## 4. Conclusions

This review summarizes information regarding the thermal conductivity of nanoporous structures. A detailed analysis of measurement techniques, review of experimental data, and a theoretical discussion on heat transfer is introduced looking for the limits in thermal conductivity.

First, an analysis of the transient and steady-state methods for measuring thermal conductivities has been performed by the description of their fundamentals and main characteristics (time-consuming, temperature range, sample size, accuracy, etc.). The advantages and drawbacks of each technique have been stated by establishing a comparison between them.

Additionally, experimental data have been provided by using techniques (TPS and heat flow meter) of both types of methods for silica aerogel composites and a PMMA sample. It has been concluded that, although both techniques showed a good correlation themselves, significantly higher values of the thermal conductivity are measured by the transient method for insulating materials. Moreover, this difference is higher when the thermal conductivity is lower, reaching differences between both techniques above 140% for thermal conductivities below 20 mW/(m K) (the values of the greatest interest). This difference is reduced when the thermal conductivity increases, being between 40 and 80% for thermal conductivities between 20–40 mW/(m K) and of 30% for thermal conductivities in the order of 70 mW/(m K). These differences suggest that the steady-state methods are the most reliable option for measuring the thermal conductivity of superinsulators, where minimum uncertainties have great relevance.

Then, data regarding porous structure and thermal conductivity of both nanocellular polymers and silica and PU aerogels have been presented, with the aim of comparison. While nanocelular polymers present some similarities regarding the porous structure with aerogels, thermal conductivity values in the literature are proved to be much higher. While silica and polyurethane aerogels present values of 10 mW/(m K) and 12 mW/(m K) respectively, nanocellular polymers do not show values below 24.8 mW/(m K).

These differences have been theoretically discussed by studying the heat transfer contributions for these structures. It has been concluded that the main differences between them lie in the gaseous conduction, since the porosity is higher for aerogels, and, mainly, in the solid conduction. The latter is promoted by the discontinuous solid phase that aerogels show, which contributes to reducing this term through a size effect on the phonon scattering.

Therefore, the development of greater insulating materials, which will allow us to explore the minimum obtainable thermal conductivity value, depends on the reduction of the bulk density and the generation of “solid interruptions” to obstruct the phonon transfer.

## Figures and Tables

**Table 1:** Main characteristics of transient and steady-state techniques in relation to the measurement of insulating materials.

	Transient Method	Steady-State Method
Type of technique	Derivative	Absolute
Power input	Pulsed power	Constant power
Accuracy	Low	High
Time consuming	Short	Long
Sample size	Small	Large

**Table 2:** Thermal conductivity measurements for insulating samples by the transient method and the steady-state method—a comparison.

Sample	Bulk Density/ kg/m^3^	TPS/ mW/(m K)	SD	Steady-State/ mW/(m K)	SD
**Silica aerogel composites**					
1	134.08	47.65	1.09	30.87	0.69
2	266.48	92.77	0.87	71.26	0.29
3	117.68	32.64	0.12	13.93	2.46
4	173.79	49.67	0.15	33.22	0.19
5	120.44	39.16	0.15	17.00	0.38
6	102.08	28.13	0.04	11.53	2.42
7	120.27	52.12	0.05	35.15	0.14
8	82.34	40.62	0.28	21.89	0.54
9	77.99	28.86	0.02	11.47	2.49
10	121.21	60.67	0.07	35.26	0.43
**PMMA nanocellular foam**					
1	403.24	79.30	1.40	51.00	0.05

**Table 4:** Super insulating silica aerogels reported in the literature.

Material	Drying ^a^	Bulk Density/ kg/m^3^	Pore Volume/ cm^3^/g	Pore Diameter/ nm	Thermal Conductivity/ mW/(m K)	Reference
Polyamide Pulp-silica aerogel composite	APD	229	n.a.	30.0	26.6	Ghica M.E. et al., 2020 [66]
Endothermic opacifier doped silica aerogel	HTSCD	n.a.	n.a.	n.a	24.6	Pang H-Q. et al., 2022 [67]
ZrO_2_^b^ fiber reinforced ZrO_2_–SiO_2_ aerogel composite	scCO_2_	230	n.a.	n.a	23.6	Hou X. et al., 2018 [68]
Silica fiber-reinforced-silica aerogel	APD	125	7.44	39.0	22.9	Torres R.B. et al., 2019 [69]
Waterglass silica aerogel	FD	n.a.	1.92	7.7	21.5	Pan Y. et al., 2018 [70]
sepiolite/silica aerogel composite	HTSCD	190	3.2	n.a.	19.7	Li X. et al., 2013 [71]
Silica aerogel powder	APD	142	2.7	12.9	19.4	Zhao S. et al., 2020 [72]
Waterglass silica aerogel powder	APD	110	2.72	12.9	19.4	Stojanovic A. et al., 2019 [73]
TEOS silica aerogel powder	APD	70	3.95	17.6	18.8	Stojanovic A. et al., 2019 [73]
PI-silica aerogel	APD	81	n.a.	n.a.	18	Liu R. et al., 2021 [74]
Pullulan/PVA-silica aerogel composite	scCO_2_	99	2.4	60	17.7	Zhao S. et al., 2018 [75]
TENCEL^®^ fibers (8 mm at 0.5 vol%) reinforced silica aerogel	scCO_2_	112	n.a.	n.a.	15.8	Jaxel J. et al., 2017 [76]
Silica Aerogel Granulate	APD	131	7.1	34	15	Huber L. et al., 2017 [77]
Vestanat^®^ EP-M 95 reinforced silica aerogel	scCO_2_	106	8.7	44.2	14.7	Iswar S. et al., 2018 [78]
silica aerogel	scCO_2_	115	8.2	38.5	14.5	Iswar S. et al., 2021 [79]
Silica-nanofribillated cellulose composite aerogel	scCO_2_	130	1.3	46	13.8	Zhao S. et al., 2015 [80]
reticulate aerogels from PTMSPMA ^c^	scCO_2_	99	n.a.	n.a	10.0	Rezaei S. et al., 2020 [81]

^a^ FD: Freeze drying; scCO_2_: supercritical drying with carbon dioxide; HTSCD: high temperature supercritical drying; APD: ambient pressure drying. ^b^ Zirconia. ^c^ Reticulate gels obtained by spinodal decomposition from 3-(trimethoxysilyl)propylmethacrylate (TMSPMA). n.a.: not available.

**Table 5:** Highly insulating polyisocianurate-polyurethane-based aerogels reported in the literature.

Material	Drying ^a^	Bulk Density/ kg/m^3^	Pore Diameter/ nm	Thermal Conductivity/ (mW/(m K))	Reference
Poly(Urethane Acrylates) and Poly(Urethane	scCO_2_	140–660	1.7–300	36–85	Bang A. et al., 2014 [93]
Polyurethane aerogels (using different isocyanates and polyols)	scCO_2_	90–760	8.3–31.9	31–103	Chidambareswarapattar C. et al., 2013 [94]
Polyurethane aerogels with MDI	scCO_2_, APD	200–240	n.a.	22–24	Rigacci A. et al., 2004 [95]
PUR-PIR ^b^ aerogels	scCO_2_	150–490	100–240	19–36	Zhu Z. et al., 2017 [96]
Polyurethane aerogels with MDI	scCO_2_	120–230	15–210	17–24	Diascorn N. et al., 2015 [97]
PIR and PUR-based aerogels	scCO_2_	150–260	11.2–17.5	15–22	Biesmans G. et al., 1998 [98]
PIR and PUR-based aerogels	scCO_2_	101–165	72–721	12–24	Merillas B. et al., 2022 [91]

^a^ scCO_2_: supercritical drying with carbon dioxide; APD: ambient pressure drying. ^b^ PUR-PIR aerogels: polyisocyanurate-polyurethane aerogels; MDI: 4,40-methylenebis(phenylisocyanate). n.a.: not available.

## Data Availability

Not applicable.
